# Parental gratification with pediatric posterior zirconia and stainless steel crowns

**DOI:** 10.6026/973206300211152

**Published:** 2025-05-31

**Authors:** Rachana Jain, Deepika Jawhari, Ritika Jain Tillani, Sakshi Malik, Brar Brar, Puneet Gupta

**Affiliations:** 1 Department of Pediatric and Preventive Dentistry Daswani Dental College and Research Center, Kota, Rajasthan, India

**Keywords:** Clinical performance, esthetic crowns, pediatric crowns, stainless steel crowns, zirconia crowns

## Abstract

The performance of posterior pediatric zirconia crowns with stainless steel crowns is of interest. Hence, 30 children (age 4-8) (17 male, 13 females) were treated with 50 pre-formed posterior primaries (25 zirconia and 25 stainless steel crowns). Crowns were evaluated for retention, marginal integrity, opposing tooth wear, gingival inflammation, and proximal contact at 3, 6, 9, and 12th month. At the end of follow-up period overall success rate with zirconia crown was 93.5% and that of stainless steel crown was 96.7%. Statistical analysis showed no difference between the groups. However, posterior preformed zirconia and stainless steel crowns showed good clinical performance. Thus, Zirconia crowns are preferred when esthetics is of prime concern for the parent and child.

## Background:

Stainless steel crowns were the choice of full coronal restoration, as they were easily available as preformed, pre trimmed and pre contoured crowns with wide range of sizes and with proven clinical efficiency [1]. The technological advances in techniques and material science led to the evolution of preformed Zirconia crowns for primary teeth. Stainless steel crowns, introduced by "Rocky Mountain" company were later improved by various manufacturers and they were used in restoring multi surface caries of primary and young permanent dentition, as post endodontic restoration abutment for space maintainer and as preventive restoration for special children. Literature evidence exposes the superiority of stainless steel crowns over conventional restorations even when used as a preventive strategy for children with medical or dental developmental disability. The only disadvantage of SSC was its unesthetic appearance [2, 3]. A survey of pediatric dentists reported that 87% of the parents are concerned about the esthetics of even posterior restorations. In addition, studies suggest that even children are more concerned about esthetics as it influences their psychological well-being and physical appearance [4, 5]. The need to meet the demand for esthetic restorations led to the introduction of open faced stainless steel crowns, pre veneered crowns, polycarboxylate crowns and strip crowns. Each of these full coronal restorations has their own advantage and disadvantage. Initially preveneered stainless steel crowns showed short term success rate but long term follow-up studies have reported frequent fracture of these crowns as a whole or a part of it [6, 7, 8- 9]. The technological advances in techniques and material science led to the evolution of preformed Zirconia crowns for primary teeth, so as to fulfil the esthetic demands, at the same time promise good durability.

Zirconia crowns are known as "Ceramic Steel" as it provides strength close to available metal crowns as well as colour similar to that of natural teeth. Pediatric zirconia crowns were introduced by EZ-pedo and became commercially available in 2008. Later preformed zirconia crowns were popularized by companies like Nusmile, Kinderkrowns, Cheng crowns, Signature crowns and many more. These preformed crowns differed with respect to size, shape, shade and pattern of retention component [10]. Advantages of preformed zirconia crowns are its esthetics, full coverage of the treated tooth, no component of the crown that might debond and potentially less technique sensitive when compared to other esthetic alternatives. The potential disadvantages include need for more tooth reduction [11] inability to crimp or contour the crown and they are also expensive. Many of the studies with preformed zirconia crowns have been confined only to the anterior teeth [12, 9]. Similarly it has been observed that impact of esthetics play an important role even in posterior primary teeth. Zirconia crowns are by far the strongest dental ceramic restoration available as preformed posterior esthetic crowns for primary dentition and very limited literature is available with regard to its efficiency and clinical performance. Therefore, it is of interest to assess and compare the efficiency of zirconia crowns with stainless steel crowns used in posterior primary teeth and also to elicit parental satisfaction for the same.

## Materials and Methods:

## Study design:

The study is a prospective clinical trial done to evaluate and compare the efficacy of preformed posterior zirconia crowns and stainless steel crowns.

## Sample size:

30 children (17 male, 13 females) were treated with 50 pre-formed posterior primaries (25 zirconia and 25 stainless steel crowns)

## Sample population:

Aged between 4-8yrs.

## Inclusion criteria:

[1] Children who required pulp therapy in primary molars indicated for full coverage restoration following pulpectomy or pulpotomy.

[2] Healthy children, free of any systemic disease or any developmental disturbances of the teeth.

[3] Children for whom consent was obtained from parents/guardians

## Exclusion criteria:

[1] Teeth with pathological root resorption or inadequate root support.

[2] Tooth with infection involving the furcation & inadequate crown structure

[3] Children with para functional habits.

## Clinical procedure:

Based on the inclusion criteria, teeth were selected and assigned into two groups

[1] Group 1-Preformed zirconia crowns

[2] Group 2-Preformed stainless-steel crowns

The evaluation of each crown restoration was assessed at the baseline which is on the same day of the procedure and the follow-up was done with an interval of 3 months till 12th month.

## Evaluation criteria:

Following criteria will be followed for evaluation: based on Parental satisfaction, (Table 1) Problems experienced by the child, (Table 2) Future treatment choice (Table 3).

## Statistical analysis:

The data was entered in the Microsoft Excel spread sheet and analyzed with WILCOXON SIGNED RANK TEST for within group comparison and MANN-WHITNEY U TEST for inter group comparison. The level of significance was set at 0.05.

## Results:

30 children (13 Female, 17 males) (Figure 1 - see PDF) were treated with 50 preformed posterior primaries (25 zirconia and 25 stainless steel crowns). A total of 25 preformed zirconia crowns and 25 SSC were followed up for a period of one year with 3 months interval. At the end of 3 months, (Table 4) one out twenty five (4%) preformed zirconia crowns showed chipping of the crown due to periapical infection (tooth extracted), whereas all the 24 stainless steel crowns (100%) were intact. Out of 25 case of SSC one case not reported for follow-up so exclude from study. At the end of 6 months, one out of 24 crowns showed complete dislodged due to occlusal harmony, with respect to zirconia crown, which was replaced by customized zirconia crown and recorded as restoration failure. All stainless steel crowns remained intact (100%). At the end of 9^th^ month, twenty three (95.83%) of zirconia crowns were intact and showed good retention, whereas one of the twenty four stainless steel crowns showed complete loss of crown due to occlusion force. At the end of 12^th^ month review twenty three (95.83%) of zirconia crowns and twenty three (95.83%) of SSC was intact. There is no significant difference in the retention ability of zirconia and stainless steel crown (p=1.000). Marginal integrity (Table 5) of the assessed crowns showed that all remaining twenty four zirconia crowns (excluding the chipped crown) showed closed margins (96%) during the 3^rd^ month follow-up period. Twenty four (100%) stainless steel crowns showed closed margins. At the end of 6 months, twenty four (100%) of SSC showed closed margins and twenty four (100%) of zirconia crowns showed closed margins. There was no alteration observed with respect to marginal integrity in preformed zirconia crowns at the end of 9^th^ and 12^th^ month follow-up. With respect to stainless steel crown, excluding the crown that dislodged due to occlusion force, all other twenty three (100%) crowns showed closed margins during the 9^th^ month follow-up. 100% success was seen in both the groups by the end of 12 months. There is no significant difference between the groups with respect to marginal integrity (p=1.000). Good proximal contact was observed throughout the follow-up period in both preformed zirconia and SSC group [Twenty four (100%) - Stainless steel and Twenty four (100%) zirconia crowns] and no opposing tooth wear (Table 6) was evident in both the groups throughout the follow-up period. Both the groups clinically showed no significant difference with respect to proximal contact and opposing tooth wear. The gingival health (Table 7) of the children showed that one tooth (4%) of the zirconia group revealed mild inflammation during the 3^rd^ month follow-up, whereas twenty four (100%) SSCs showed no signs of inflammation. At 6^th^ month review, two of the zirconia crowns showed moderate inflammation with bleeding on probing and twenty four (100%) of Stainless steel crowns showed no inflammation. 100%success was observed in both the groups with respect to gingival health at the end of 12^th^ month follow-up. There is no significant difference between zirconia crowns and stainless steel crowns with respect to gingival status (p=0.317). Parental satisfaction (Table 8) was obtained from 25 parents with the help of 5 point likert type scale regarding size, shape, colour, durability, impact on oral health and appearance along with it problems associated with crown placement such as sensitivity, bleeding around gums and food lodgment as well as future treatment considerations were elicited with a dichotomous 'Yes/No' questionnaire. The majority of the parents were satisfied with size and shape of crowns with a mean score of 4.3 on l5-point likert scale for stainless steel crowns and 4.8 with zirconia crowns. All the parents were satisfied with the colour of zirconia crowns (100%) with the mean score of 5, whereas only 36% were not satisfied with colour of stainless steel crowns, 32% were neutral and 32%reported no objection with colour of stainless steel crowns. 96% of the parents were satisfied with durability of crowns in both the groups, where 4% of them reported complete loss of crowns atleast once. Majority of the parents reported improvement of child's oral health after placement of crowns in both the groups, with a mean of 4.8 (96%). Overall satisfaction with stainless steel crowns were good with the mean score of 4.4 and washing her with zirconia crown with a mean of 4.9 [p=0.03]. Problems experienced by the children were evaluated with a 'Yes/No' question. Parents reported that there were no difficulties such as sensitivity, bleeding around crown and food lodgment with stainless steel crowns whereas 8% of them reported bleeding around the crown with zirconia crowns. No problem was reported with respect to sensitivity and food lodgment in zirconia group. All the evaluated crowns from both the groups showed 100% good marginal adaptation. Majority of the parents in both the groups SSCs and zirconia crowns) were satisfied with the crowns. Mild bleeding (8%) was reported in zirconia group. Parents were ready to recommend both the crowns with preference for zirconia crowns because of its colour being similar to that of natural teeth.

## Statistical analysis:

The data was entered in the Microsoft Excel spread sheet and analyzed with WILCOXON SIGNED RANK TEST for within group comparison and MANN-WHITNEY U TEST for inter group comparison. The level of significance was set at 0.05.

## Discussion:

Table 9 full coronal restorations have become the prevalent part of rehabilitation of the children affected with early childhood caries. Multiple options have been tried with each one showing varied clinical performance. Change in life style, more opportunities to socialize and role of media plays a role in exposing the children to a concept of ideal beauty at very young age. This has showed impact on their concerns about esthetics which is similar to that of adults. The same principle applies in terms of restorations to be placed on their teeth [5]. With the concept of esthetics gaining interest among parents and children, preformed zirconia crowns were becoming much more popular [12, 13]. But very limited literature is available regarding their performance. Keeping the above fact in mind the present study was conducted to assess and compare the clinical performance and parental satisfaction of pediatric zirconia crowns with that of stainless steel crowns which were the most commonly used crowns for posterior primary teeth [16, 14]. In this study a total of 50 crowns (25 zirconia and 25 stainless steel) were placed in 30 patients following pulp therapy. Of which 49 crowns were available for 12 months follow up (24 SSC and 25 Zirconia crowns). The clinical evaluation criteria were similar to the one used by, Holsinger *et al.* and Abdulhadi *et al.* The parental satisfaction of the crowns were obtained through a survey with a set of dichomotous 'Yes/No' questions and 5 point likert type scale adopted from other studies [8, 15 and 13, 18]. In this study performed kinder crowns were used due to the availability of varied sizes, better retentive features and a polished surface to reduce opposing tooth wear. According to the manufacturers guidelines, resin modified glass ionomer (3M ESPE Rely X) were used to lute the crowns. Preformed Stainless Steel Crowns were luted with type 1 GIC (GC Type 1 GIC) as it is the most commonly recommended cement for luting stainless steel crowns [8]. The present study showed 95.83 % retention with stainless steel crown. Loss of stainless steel crown 4.3% during 9th month follow up could be attributed to loss of cement. Micro structural porosity or the voids in the set cement can progressively cause crack propagation due to occlusal forces finally leading to cement loss over a period of time [14, 16]. This could be the attributing factor for crown dislodgement at the end of 9th month review. The crown was re-cemented and reviewed till end and it was found to be intact. 95.83% of zirconia showed complete retention of crowns. 3.2% of zirconia crowns showed chipping of the crown and 3.2% showed complete loss of crown at 3rd month follow up. The chipping of the crown could be attributed to the occlusal forces delivered while seating the crown or due to mild occlusal disharmony as a result of inability to trim the cuspal patterns of these crowns. The complete loss of crown in zirconia group could be as a result of inadequate tooth structure to achieve sub-gingival preparation, functional cusp of opposing tooth due to inadequate moisture control while cementing or due to loss of cement. The crown which showed complete loss was recorded as restoration failure and it was replaced by custom made zirconia crown and not assessed for other parameters. These results are contrary to the findings of Gayathri *et al.* who showed 100% retention of zirconia crowns [18]. The cements used in the present study to lute the crowns were different and that might have influenced the bond strength which in turn could have influenced the retention of the crowns [9, 3 and 11]. The crowns other than those which showed retention failure had good marginal integrity in both the groups (100% with SSC and 96.6%) with zirconia crowns which was higher than that of Holsinger *et al.* [12] showed 86% closed margins with zirconia crowns. The fine feather margins could have contributed in good adaptation of crown to the tooth structure irrespective of excessive tooth preparation. Stainless steel crowns are easy to contour and crimp, making them adapt well to the prepared tooth structure. All the stainless steel crowns evaluated for gingival inflammation showed healthy gingiva without any inflammation, whereas three children showed visible mild to moderate gingival inflammation in the zirconia group which was resolved during 9th month review. Gingival inflammation can be attributed to the generalized plaque accumulation and lack of motivation which was improved with oral prophylaxis and oral health education and motivation. Results showed that gingival health was better in teeth restored with stainless steel crowns than those which treated with zirconia crowns during 3 and 6 months follow-up .Reversal of the condition was observed at the end of 9^th^ month review which was achieved through oral prophylaxis and oral hygiene education. Our results were contrary to the findings of Abduhadi *et al.* that showed better gingival health with zirconia crowns (100%) when compared to stainless steel (75%). He stated that shaping of metal borders improperly as well as adhesive residues in the sulcus is the reason for gingival problems in case of stainless steel crowns, whereas glazed and polished surface of the zirconia crowns resulted in less plaque accumulation and good gingival health. Both the groups showed no opposing tooth wear. This was contrary to the findings of studies Walia *et al.* [9]. Donovon *et al.* suggested opposing tooth wear occurs if the crown surface is improperly glazed [17]. In the present study, Kinder krown were used which were manufactured based on nano technology with highly polished occlusal surface, resulting in no occlusal wear of opposing tooth. Till date no case of opposing tooth wear has been reported with stainless steel crowns because of its ability to adapt to the occlusal forces. The result of this study suggests that both pediatric posterior zirconia crowns and stainless steel crowns maintained, marginal integrity with no opposing tooth wear. The results highlight the role of oral hygiene maintenance and health education on the gingival health with respect to crowns. The results also indicate the necessity of acquiring skills in preparing and placing the crowns to achieve best performance of the crowns. Parental satisfaction obtained with a 5-point likert scale and an 'Yes/No' question revealed good satisfaction with respect to size and shape of both the crowns with better results with zirconia crowns (4.9 MEAN SCORE). The values were slightly higher than the findings of Gayathri *et al.* (4.8) [18]. With respect to colour, zirconia out performs stainless steel (2.9) with a mean score of 5. Both zirconia and stainless steel crowns performed similar with respect to parents perception of durability with a mean score of 4.8 which was slightly higher than the values obtained by Walia *et al.* (4.7) [9]. Overall satisfaction with zirconia crown (4.9) was better than the stainless steel crowns (4.4). Limitations of the study were inability to follow split mouth design with only one year follow-up. Further studies are recommended following a split mouth design, testing different brands of commercially available zirconia crowns with varied level of polish, gloss and morphological variations for a longer duration to get more valuable information on the clinical performance of preformed zirconia crowns. Further studies are recommended to test the crowns efficiency in varied clinical scenarios, such as crowded dentition, dentition with occlusal variation and placement of multiple crowns. Both stainless steel crowns and zirconia crowns are an excellent choice for posterior primary teeth as their clinical performance and parental satisfaction was high. However stainless steel crowns performed better in terms of retention. But zirconia crowns can be the choice of post endodontic restoration when esthetics is of prime concern.

## Conclusion:

Pediatric zirconia (93.5%) and stainless steel crowns (96.7%) showed good clinical success rate with no significant difference between the two groups (p=0.317). Crown retention, marginal integrity, accumulation of plaque and gingival health were better with stainless steel crown compared to zirconia crowns but not to the significant level (p=1.000). Parental satisfaction was significantly higher with zirconia (96%) crowns with (p=0.03). Clinical performance of crowns was good, so choice of the crowns during treatment plan can be made based on the demands of the parents and the clinical scenario.

## Figures and Tables

**Table 1:** Parental satisfaction

**Category**	**Scoring criteria**
Size	Score 1-very dissatisfied
Shape	Score 2- dissatisfied
Colour	Score 3- neutral
Durability	Score 4- satisfied
Impact on oral health & appearance	Score 5-very satisfied
Over-all satisfaction	

**Table 2:** Problems experienced by the child

**CATEGORY**	
Bleeding around crown	Yes/ no
Sensitivity	Yes/ no
Food lodgment	Yes/ no

**Table 3:** Future treatment choice

**Category**	**Stainless steel crown**	**Zirconia crown**
	yes/ no	yes/no

**Table 4:** Retention of stainless steel and zirconia crown at 3, 6, 9 and 12th month follow-up visit

**Category**	**Stainless steel crown**				**Zirconia crowns**			
CROWN RETENTION	3 M (24)	6M (24)	9M (24)	12M (24)	3 M (25)	6M (24)	9M (24)	12M (24)
Intact	24 (100%)	24 (100%)	23 (95.83%)	23 (95.83%)	24 (96%)	23 (95.83%)	23 (95.83%)	23 (95.83%)
Chipped					1 (4%)			
Complete Loss			1 (4.3%)					

**Table 5:** Marginal integrity of stainless steel and zirconia crown at 3, 6, 9 and 12th month follow-up visit

**Category**	**Stainless steel crown**				**Zirconia crowns**			
MARGINAL INTEGRITY	3 M (24)	6M (24)	9M (24)	12M (24)	3 M (25)	6M (24)	9M (24)	12M (24)
Closed	24	24	23	23	24	24	24	24
Open					1			

**Table 6:** Opposing tooth wear caused by stainless steel and zirconia crowns at 3, 6, 9, 12th month follow-up visit

**Category**	**Stainless steel crown**				**Zirconia crowns**			
Opposing Tooth Wear	3 M (24)	6M (24)	9M (24)	12M (24)	3 M (25)	6M (24)	9M (24)	12M (24)
Wear								
No Wear	Present							

**Table 7:** Gingival inflammation with stainless steel and zirconia crowns at 3, 6, 9 and 12th month follow-up visit

**Category**	**Stainless steel crown**				**Zirconia crowns**			
Gingival inflammation	3 M (24)	6M (24)	9M (24)	12M (24)	3 M (25)	6M (24)	9M (24)	12M (24)
No inflammation	24/24 (100%)	24/24 (100%)	23/23 (100%)	23/23 (100%)	24/25 (96%)	22/24 (91.60)	24/24 (100%)	24/24 (100%)
Mild					1			
Moderate						2		
Severe								

**Table 8:** Parental satisfaction

**CATEGORY**	**STAINLESS STEEL CROWN Mean score**	**ZIRCONIA CROWNS Mean score**
Size and Shape	4.3	4.8
Colour	36% were not satisfied	5
	32% were neutral	
	32% reported no objection	
Improvement of child's oral health after placement of crowns	4.8	4.8
Overall Satisfaction	4.4	4.9

**Table 9:** Different study comparison with crown retention, marginal integrity, opposing tooth wear, gingival inflammation, and parent satisfaction

**Study**	**CROWN RETENTION**		**MARGINAL INTEGRITY**		**OPPOSING TOOTH WEAR**		**NO GINGIVAL INFLAMMATION**		**PARENTAL SATISFACTION**	
**At 12 month follow up**									**Mean score**	
	SSC %	ZRC %	SSC %	ZRC %	SSC %	ZRC %	SSC %	ZRC %	SSC %	ZRC %
Present study	95.8	95.8	100	100	absent	absent	100	100	4.4	4.9
Gayathri *et al.*	100	96.6	100	100	absent	absent	100	100	4.2	4.8
Holsinger *et al.*				86						
Abduhadi *et al.*							75	100		
Walia *et al.*					Present	Present				
